# Pathological Specimens of Teeth

**Published:** 1870-02

**Authors:** 


*Pathological Specimens of Teeth.*—We are indebted to Dr. Jno. C. Story, of Emory, Virginia, for several interesting specimens for the Museum of the Baltimore College of Dental Surgery. The friends of the College can greatly aid the cause of education by contributions of this character, for which due credit will be given, and the name of the donor attached to the contribution.

